# Metabolic Syndrome: An Indicator of Complicated Gall Stone Disease?

**DOI:** 10.7759/cureus.3659

**Published:** 2018-11-30

**Authors:** Monis J Ahmed, Rasheeqa Mahmood, Roshane S Rana, Muhammad T Pirzada, Jahanzaib Haider, Sheeraz S Siddiqui, Shams N Alam

**Affiliations:** 1 Surgery, Mediclinic City Hospital, Dubai, ARE; 2 Surgery, Jinnah Medical College Hospital, Karachi, PAK; 3 Urology, Agha Khan University Hospital, Karachi, PAK; 4 Surgery, Dow University of Health Sciences, Karachi, PAK; 5 Surgery, The Indus Hospital, Karachi, PAK

**Keywords:** cholelitiasis, metabolic syndrome, complicated gallstone disease

## Abstract

Background

Gallstone disease is a common surgical entity worldwide and accounts for a major portion of hospital admissions and surgeries. Metabolic syndrome is diagnosed when three of the following medical conditions are positive: central obesity, high blood pressure, increased fasting glucose levels, low high-density lipoprotein (HDL) levels and high serum triglycerides.

Objective

To compare the frequency of metabolic syndrome in patients with uncomplicated gallstone disease and complicated gallstone disease.

Study design

Observational, cross-sectional study.

Methodology

A total of 104 patients, above age 18 years, visiting the outpatient department* *(OPD) or the emergency department, diagnosed as having gallstone disease. The study was conducted in surgical unit VI, civil hospital Karachi from June 2014 to June 2015. Patients’ demographics, abdominal waist circumference, blood pressure, serum fasting blood sugar, triglyceride level and HDL levels were recorded. Final outcome was labeled as presence or absence of metabolic syndrome. Presence of metabolic syndrome was compared in patients with complicated gallstone disease as well as in patients with uncomplicated gallstone disease. Chi square test was used to detect statistical significance and a p-value of <0.05 was taken as significant.

Results

The ages were comparable between the two groups, that is, the complicated and uncomplicated gallstone disease at 42.42 +/- 12.15 years in the former and 39.24 +/- 10.41 years in the latter group. Metabolic syndrome was more predominant in the complicated arm 40.38% when compared to uncomplicated arm 25% but it was not significant statistically with a p-value of 0.2.

Conclusion

Metabolic syndrome is associated with complicated gallstone disease though this study failed to reach statistical significance due to small sample size, it re-enforces the findings of previous studies. It is an easily assessable and useful measure to predict complications associated with gall stone disease.

## Introduction

Gallstone disease is a common surgical entity worldwide [1] and accounts for a major portion of hospital admissions and surgeries and is a major cause of morbidities in both the developed and the developing nations. Though mostly prevalent in the females, the male population also accounts for a significant portion of the affected population. The most common type of gallstones are the cholesterol stones [2], the pathogenesis of which is multifactorial. The complications of gall stones range from acute cholecystitis to perforated gall bladder and pancreatitis.

Metabolic syndrome is summarized as a problem in energy utilization and storage. It is diagnosed when three of the following medical conditions are positive: central obesity, high blood pressure, increased fasting glucose levels, low high-density lipoprotein (HDL) levels and high serum triglycerides [3]. It was first described in 1921 and was modified multiple times [4].

Incidental cholelithiasis has been traditionally discouraged to be treated surgically, for not every patient becomes symptomatic [5] and the risks of complications from the procedure and the cost of healthcare outweigh the benefit. The number of cases with incidental findings of cholelithiasis is increasing due to easy availability and noninvasiveness of ultrasonography. The risk of the disease becoming symptomatic, increases with every year after the diagnosis [6] and then once symptomatic, the risk of complications is high [7]. The need for a marker that is able to predict the occurrence of complications is still unanswered.

The association of metabolic syndrome and gallstones has been highlighted by a number of studies [4]. However, a recent study identified the presence of metabolic syndrome as a risk factor for complicated gallstones disease [8]. This finding is significant as those patients who have incidental cholelithiasis together with metabolic syndrome may benefit from prophylactic cholecystectomy in order to reduce the morbidity associated with the complicated gallstone disease.

The aim of this study was to compare the prevalence of metabolic syndrome in uncomplicated and complicated cases of gallstones. This will provide a local perspective and local protocols could be developed so as to reduce the morbidity of complicated gallstone disease by offering prophylactic cholecystectomy in these patients. The objective of this study was to compare the frequency of metabolic syndrome in patients with uncomplicated gallstone disease and complicated gallstone disease.

## Materials and methods

This descriptive study was performed after the approval of the institutional authority of the university hospital. Uncomplicated gallstone disease was diagnosed on the basis of ultrasonographic evidence of gall stones with no evidence or history of an episode of acute cholecystitis and pancreatitis. Complicated gallstone disease was diagnosed on the basis of ultrasonographic evidence of acute cholecystitis, or history of an episode of acute cholecystitis, or acute biliary pancreatitis or the active presence of these diseases.

Patients were labelled as having the metabolic syndrome if they fulfilled three or more of the following criteria: waist circumference greater than 102 cm for men, greater than 88 cm for women; diagnosed with hypertension or receiving antihypertensive medication, or two blood pressure measurements exceeding 130/85 mmHg; diagnosed with diabetes mellitus or receiving antidiabetic treatment, or a fasting blood glucose level of greater than 110 mg/dl; HDL-C level lower than 40 mg/dl for men, lower than 50 mg/dl for women; and triglyceride levels above 150 mg/dl.

Patient selection

A total of 112 patients, above age 18 years, visiting the outpatient department (OPD) or the emergency department, diagnosed as having gallstone disease on the basis of ultrasonography by consultant general surgeon, fulfilling the inclusion criteria were included in the study. Patients who were taking statins or fibrates and who did not give consent were excluded from the study. The sample size was calculated by using openepi.com, version II, open source calculator taking the prevalence of metabolic syndrome in uncomplicated gallstone disease to be 22% and that in complicated cases to be 47% [8]. Study was conducted in surgical unit VI, Civil hospital Karachi from June 2014 to June 2015. Informed consent was taken from each patient for participating in the study. After obtaining complete history and conducting detailed physical examination all baseline investigations including serum fasting glucose and triglycerides and HDL levels were sent. The patients’ abdominal circumference at the umbilicus level was measured and blood pressure was recorded on two separate occasions at least six hours apart.

A proforma was used to record patients demographic like age, gender and hospital registration number along with the diagnosis that is complicated or uncomplicated gallstone disease. It also included the abdominal waist circumference, blood pressure, serum fasting blood sugar, triglyceride level and HDL levels. Final outcome was labeled as presence or absence of metabolic syndrome. Out of the total patients, eight patients’ data was incomplete and they were excluded from the study, and the total numbers of patients considered were 104.

Data analysis

Mean and standard deviation were computed for numerical variables like age, serum fasting blood glucose, abdominal circumference, blood pressure, triglyceride and HDL levels. Frequency and percentages were used for categorical data like gender, complicated and uncomplicated gallstone disease, and presence or absence of metabolic syndrome. Presence of metabolic syndrome was compared in the two groups and chi square test was used to detect statistical significance and a p-value of <0.05 was taken as significant.

## Results

The ages were comparable between the two groups, that is, the complicated and uncomplicated gallstone disease at 42.42 +/- 12.15 years in the former (n = 52) and 39.24 +/- 10.41 (n = 50) years in the latter group. The Mean +/- SD waist circumference in the complicated group was 89.5 +/- 10.59 cm (n = 51) in the complicated arm and 90.35 +/- 16.03 cm (n = 52) in the uncomplicated arm. The Mean +/- SD HDL level in the complicated group (n = 44) was 30.66 + 14.59 mg/dl in the complicated arm and 38.41 +/- 12.52 mg/dl in the uncomplicated arm (n = 41). The Mean +/- SD serum triglyceride level in the complicated group was 116.28 +/- 58.38 mg/dl in the complicated arm (n = 50) and 118.67 +/- 65.43 mg/dl in the uncomplicated arm (n = 49). The Mean +/- SD fasting blood sugar (FBS) level in the complicated group was 115.39 +/- 59.89 mg/dl in the complicated arm (n = 51) and 97.29 +/- 21.51 mg/dl in the uncomplicated arm (n = 48). The Mean systolic blood pressure (BP) in uncomplicated group was 117.08 +/- 12.1 mmHg, Mean diastolic BP of uncomplicated group was 74 +/- 8.5 mmHg (n = 46). The Mean systolic BP of complicated group was 116.72 +/- 16.61 mmHg, Mean diastolic BP of complicated arm was 72.93 +/- 11.4 mmHg (n = 50), p-value for systolic BP was 0.90 and p-value for diastolic BP was 0.60 (Table 1).

**Table 1:** Patient Parameters. HDL: High-density lipoprotein; FBS: Fasting blood sugar.

	Complicated	Uncomplicated	p-Value
Waist (cm)	89.5 ± 10.59 (n = 51)	90.35 ± 16.03 (n = 52)	0.75
HDL (mg/dl)	30.66 ± 14.59 (n = 44)	38.41 ± 12.52 (n = 41)	0.01
Triglycerides (mg/dl)	116.28 ± 58.38 (n = 50)	118.67 ± 65.43 (n = 49)	0.84
FBS (mg/dl)	115.39 ± 59.89 (n = 51)	97.29 ± 21.51 (n = 48)	0.05

Metabolic syndrome was more predominant in the complicated arm 40.38% when compared to uncomplicated arm 25%but this difference did not reach a statistical significance with a p-value of 0.2.

## Discussion

The association of obesity to a variety of different diseases is a common ground for extensive research in public health initiatives. Abdominal obesity results in an inefficiency of serum insulin to manifest its effects of carbohydrate and fat metabolism. This phenomenon is a proposed precursor to the development of diabetes in type II diabetic patients. The same insulin ‘resistance’ that the cells of an obese patient demonstrate also has effects on the cardiovascular system leading to its diseased state [9]. The association of these two class of diseases, that is diabetes and cardiovascular, with a number of metabolic derangements and the linkage between the latter two and obesity led to the proposition of the metabolic syndrome aka syndrome X, the deadly quartet, insulin resistance syndrome [10]. A number of different criteria are being used for the establishment of metabolic syndrome, amongst which the National Cholesterol Education Program (NCEP) Adult Treatment Panel III (ATP III) is the most common [11].

Cholelithiasis or gallstones are one of the most common surgical diseases encountered throughout the world though the frequency and prevalence vary from region to region. The burden of the disease in the USA is estimated to be 10–15% of the population [12]. Though no specific data is available for the Pakistani population one local study puts the surgical incidence to be 9.03% [13]. Another study from the neighboring India also gives nearly the same prevalence in their population [14]. The pathogenesis of the disease is multi-factorial and the factors include ethnicity, genetics, age, female gender, obesity, dyslipidemia, rapid weight loss, total parenteral nutrition and socioeconomic status amongst others [15].

Sonography has become an increasingly well available and relatively cheap mode of investigation into abdominal problems. With the number of people having asymptomatic gallstones to be as high as 80% [16], the numbers of patients who are being diagnosed with these stones are ever increasing. This leads to a dilemma for the surgical community as routine surgery is not the treatment option for this subtype of patient population. The current indications of surgery in asymptomatic gallstones include biliary cancer, and those with choledocholithiasis, sickle cell disease, gallstones larger than 3 cm, or significant immunosuppression [17].

Only 10–25% of the patients who are found to have incidental/asymptomatic gallstones ever become symptomatic in their life time [16]. That translates into the fact that around 75% of these patients will undergo an unnecessary procedure. This not only means putting the patient in unnecessary risk for morbidity and mortality but for a country like Pakistan and even the developed nation it translated into an extremely high cost of healthcare incurred. Though the above mentioned indications do narrow down to the patients who are most likely to benefit from surgery it is still difficult to predict which patients are most likely to develop symptomatic disease in the future.

Metabolic syndrome status is an easily assessable measure that can be performed in an outpatient setting. Metabolic syndrome has been stipulated to be an independent risk factor for the development of gallstones itself [4,18]. We carried out this study to evaluate its association with complicated gallstones. The results of this study show that the patients who have complicated gallstone disease have a higher incidence of metabolic syndrome as compared to those with uncomplicated disease. However, this difference did not reach statistical significance in our study. Although the finding of this study is comparable to other studies of similar nature with regards to higher prevalence of metabolic syndrome in complicated gallstones, e.g. Ata et al. [8]^ ^also observed the same pattern in their patient population. Our results need to be carefully aligned with the fact that we were unable to reach appropriate power for the study and were including a smaller sample size. The prevalence of metabolic syndrome was still overall more in the complicated group in this study.

Patient education and involvement in decision making is without question the center point of modern medical practice. It is very much needed to give them a fair idea of how likely are they to develop symptoms in the future. The results of these two studies indicate the utility of evaluation of the metabolic syndrome status of the patient to answer this viable and important question. In the light of the finding of this study it can be indicated that the presence of metabolic syndrome in a patient with incidental gall stones can be an indication for offering ‘prophylactic’ surgery as they have an increased risk to develop complicated gallstone disease. This will help reduce the number of unnecessary procedures and not only save patients from risk of surgical complications but also reduce the burden on and cost of healthcare.

The similarity of association and re-demonstration of the findings in this study gives additional support to the use of metabolic syndrome status in assessment of patients with silent cholelithiasis. Still, as both the primary studies are on small scale the evaluation of the findings in multicentric trials needs to be conducted for providing further data.

## Conclusions

Metabolic syndrome is associated with complicated gallstone disease, though this study failed to reach statistical significance due to small sample size, it re-enforces the findings of previous studies. It is an easy assessable and useful measure to predict complications associated with gallstone disease.
